# Changes in lifestyle risk factors: health and economic impact as estimated by the population based RHS model

**DOI:** 10.1186/1472-6963-14-S2-P41

**Published:** 2014-07-07

**Authors:** Inna Feldman, Dmitry Grigorovich, Pia Johansson

**Affiliations:** 1 Dept. of Women’s and Children’s Health, Uppsala University, Uppsala, Sweden; 2 Financial University, Moscow, Russia; 3 Stockholm County Council, Stockholm, Sweden

## Background

The ability to estimate societal cost-savings given a change in population lifestyles is of interest to health promotion specialists and decision makers. A population-based model named Risk factors, Health and Societal Costs (RHS) was developed to simulate changes in incidence and related societal costs of several chronic diseases after five to ten years, following assumed changes in four common risk factors for disease: obesity (BMI>30), daily tobacco smoking, lack of physical activity and risky consumption of alcohol.

## Materials and methods

The RHS model is based on relative risks (RR) and potential impact fractions (IF) that simulate the changes in disease incidence of reducing the exposure to risk factors [1]. Relative risks as well as disease-specific QALY and DALY weights were collected from international publications. Swedish national registers were used to retrieve incident cases and disease-specific medical care costs, while local authority costs for care and sickness insurance expenses were estimated via a Swedish study [2]. The health gains are calculated as decreased number of incident cases of disease, increased health-related quality of life (QALYs) and decreases in disability (DALYs).

## Results

The scenarios, which assume a certain reduction in population risk factor prevalence, show that considerable health gains and savings in societal costs can arise from modest changes in population lifestyle habits. As an example, a 1% reduction in prevalence in daily smoking under five years among Stockholm county population is estimated to lead to health gain of 64 QALYs and societal savings (health care, municipality care and sickness insurance) of 13 million Swedish krona (1.2 million GBP).

## Conclusion

The RHS model estimates future cases of illness and related societal costs due to lifestyle risk factors in the population. By creating scenarios with assumed changes in risk factors, the model can estimate potential health gains and societal costs savings, which can be used as relevant arguments in discussions with decision-makers for a more health-promoting health care system.
